# *LMO1* polymorphisms reduce neuroblastoma risk in Chinese children: a two-center case-control study

**DOI:** 10.18632/oncotarget.20018

**Published:** 2017-08-07

**Authors:** Jiao Zhang, Huiran Lin, Jiaxiang Wang, Jing He, Da Zhang, Pan Qin, Lin Yang, Lizhao Yan

**Affiliations:** ^1^ Department of Pediatric Surgery, The First Affiliated Hospital of Zhengzhou University, Zhengzhou 450052, Henan, China; ^2^ Department of Pediatric Surgery, Guangzhou Institute of Pediatrics, Guangzhou Women and Children’s Medical Center, Guangzhou Medical University, Guangzhou 510623, Guangdong, China; ^3^ Animal Experimental Management Center, Public Technology Service Platform, Shenzhen Institutes of Advanced Technology, Chinese Academy of Sciences, Shenzhen 518055, Guangdong, China

**Keywords:** LMO1, neuroblastoma, GWAS, polymorphism, susceptibility

## Abstract

Previous genome-wide association and validation studies suggest that *LIM domain only 1 (LMO1)* gene polymorphisms affect neuroblastoma susceptibility. In this work, we used Taqman methodology to genotype four *LMO1* polymorphisms (rs110419 A > G, rs4758051 G > A, rs10840002 A > G and rs204938 A > G) in 118 neuroblastoma cases and 281 controls from Northern China. Odds ratios (ORs) and 95% confidence intervals (CIs) were used to evaluate the association. We found that rs4758051 G > A was associated with a decreased neuroblastoma risk (AA vs. GG: adjusted OR = 0.28, 95% CI = 0.13–0.62; AG/AA vs. GG: adjusted OR = 0.62, 95% CI = 0.40–0.97; AA vs. GG/AG: adjusted OR = 0.33, 95% CI = 0.15–0.69). Likewise, carrying the rs10840002 G allele was also associated with a decreased neuroblastoma risk in this Northern Chinese population. In a combination analysis using Southern and Northern Chinese populations, we found that those carrying the rs110419 G, rs4758051 A or rs10840002 G allele were at decreased neuroblastoma risk, and this finding was supported by a false-positive report probability analysis. These results further verify that *LMO1* polymorphisms are protective against neuroblastoma. Case-control studies with larger samples and using other ethnicities are still needed to confirm our conclusion.

## INTRODUCTION

Neuroblastoma is a common solid tumor derived from primordial sympathetic neural precursors and has complicated clinical manifestations [1]. Around the world, it ranks as the third leading cause of cancer-related death in children [2]. In China, neuroblastoma accounts for nearly 10% of childhood tumors, and its incidence is about 7.7 cases per million [3]. Despite great achievements in multimodality treatment, the 5-year survival rate for neuroblastoma remains at less than 40% [4]. Moreover, due to their chronic health conditions, survivors have difficulty finding marriage partners and employment [5]. Thus, neuroblastoma remains a great burden for the affected children and for their families and society [6].

The etiology of neuroblastoma is not yet fully understood, and the risk factors affecting the susceptibility to neuroblastoma have not been well documented [7]. Epidemiological studies suggest children may be more susceptible to neuroblastoma if their parents were exposed to environmental risk factors, including radiation sources, wood dust and hydrocarbons [8, 9]. However, most children whose parents are exposed to environmental risk factors do not develop neuroblastoma [10]. Mounting evidence suggests genetic polymorphisms may somehow influence the predisposition to neuroblastoma [11–15].

The *LIM domain only 1* (*LMO1*) gene is located on chromosome 11p15 and encodes an intertwining LIM-only transcriptional regulator [16]. *LMO1* is a member of the *LMO* gene family [17, 18] and is highly expressed in bone marrow and the nervous system [19], though it was first identified at human T cell acute lymphoblastic leukemia chromosomal translocations [20]. LMO1 protein has been implicated in the initiation and development of several cancers [18]. In addition, *LMO1* gene single nucleotide polymorphisms (SNPs) reportedly affect susceptibility to acute lymphoblastic leukemia [21] and neuroblastoma [16].

We previously investigated the association between *LMO1* polymorphisms and neuroblastoma risk in a Southern Chinese population [22]. Given the likely genetic variation across different regions, the respective roles of genetic factors in neuroblastoma risk may differ. Therefore, to further confirm the relationship between *LMO1* polymorphisms and neuroblastoma risk, we performed the current hospital-based case-control study using subjects from Northern China.

## RESULTS

### Population characteristics

The demographic characteristics of the included 118 cases and 281 controls are summarized in Supplementary Table 1. No significant differences were observed in the age (*P* = 0.189) or gender (*P* = 0.196) distribution between cases and controls. According to the INSS criteria [23], 15 (12.82%) patients were classified as stage I, 31 (26.50%) as stage II, 19 (16.24%) as stage III, 49 (41.88%) as stage IV, and 3 (2.56%) as stage 4s neuroblastoma. Among these, 89 (75.42%) tumors originated in the adrenal gland, 19 (16.10%) in the mediastinum, and 10 (8.47%) and other regions.

### *LMO1* polymorphisms and neuroblastoma risk in Northern Chinese children

The genotype frequencies of the four *LMO1* polymorphisms and their associations with neuroblastoma risk are listed in Table 1. Among the controls, all four tested SNPs were in Hardy-Weinberg equilibrium (all *P*_HWE_ > 0.05). Moreover, the analyses indicated that carrying the rs4758051 A allele had a protective effect against developing neuroblastoma (AA vs. GG: adjusted odds ratio (OR) = 0.28, 95% confidence interval (CI) = 0.13–0.62, *P* = 0.002; AG/AA vs. GG: adjusted OR = 0.62, 95% CI = 0.40–0.97, *P* = 0.035; AA vs. GG/AG: adjusted OR = 0.33, 95% CI = 0.15–0.69, *P* = 0.003). Similarly, we found that carrying the rs10840002 G allele was associated with a decreased risk of neuroblastoma (GG vs. AA: adjusted OR = 0.42, 95% CI = 0.21–0.85, *P* = 0.016; GG vs. AG/AA: adjusted OR = 0.48, 95% CI = 0.26–0.91, *P* = 0.025). However, we failed to detect an association between the rs110419 A > G or rs204938 A > G polymorphism and neuroblastoma risk, whether or not adjusted for age and sex.

**Table 1:** Association of LMO1 polymorphisms with neuroblastoma susceptibility in children from Henan province

Genotype	Cases (*N* = 118)	Controls (*N* = 281)	*P* ^a^	Crude OR (95% CI)	*P*	Adjusted OR (95% CI)^b^	*P*^b^
rs110419 (HWE = 0.677)							
AA	47 (39.83)	86 (30.60)		1.00		1.00	
AG	54 (45.76)	142 (50.53)		0.70 (0.43–1.12)	0.134	0.69 (0.43–1.11)	0.122
GG	17 (14.41)	53 (18.86)		0.59 (0.31–1.13)	0.109	0.57 (0.30–1.09)	0.090
Additive			0.179	0.75 (0.55–1.03)	0.074	0.74 (0.54–1.01)	0.060
Dominant	71 (60.17)	195 (69.40)	0.074	0.67 (0.43–1.04)	0.075	0.65 (0.42–1.03)	0.064
Recessive	101 (85.59)	228 (81.04)	0.286	0.72 (0.40–1.31)	0.287	0.71 (0.39–1.28)	0.253
rs4758051 (HWE = 0.946)							
GG	50 (42.37)	88 (31.32)		1.00		1.00	
AG	59 (50.00)	138 (49.11)		0.75 (0.47–1.19)	0.228	0.76 (0.48–1.21)	0.252
AA	9 (7.63)	55 (19.57)		**0.29 (0.13–0.63)**	**0.002**	**0.28 (0.13–0.62)**	**0.002**
Additive			0.006	**0.60 (0.43–0.84)**	**0.003**	**0.60 (0.43–0.83)**	**0.002**
Dominant	68 (57.63)	193 (68.68)	0.034	**0.62 (0.40–0.97)**	**0.035**	**0.62 (0.40–0.97)**	**0.035**
Recessive	109 (92.37)	226 (80.43)	0.003	**0.34 (0.16–0.71)**	**0.004**	**0.33 (0.15–0.69)**	**0.003**
rs10840002 (HWE = 0.620)							
AA	42 (35.59)	78 (27.76)		1.00		1.00	
AG	62 (52.54)	144 (51.25)		0.80 (0.50–1.29)	0.360	0.81 (0.50–1.31)	0.389
GG	14 (11.86)	59 (21.00)		**0.44 (0.22–0.88)**	**0.021**	**0.42 (0.21–0.85)**	**0.016**
Additive			0.064	**0.69 (0.50–0.95)**	**0.025**	**0.68 (0.50–0.94)**	**0.021**
Dominant	76 (64.41)	203 (72.24)	0.119	0.70 (0.44–1.10)	0.120	0.69 (0.44–1.10)	0.119
Recessive	104 (88.14)	222 (79.00)	0.031	**0.51 (0.27–0.95)**	**0.034**	**0.48 (0.26–0.91)**	**0.025**
rs204938 (HWE = 0.687)							
AA	77 (65.25)	168 (59.79)		1.00		1.00	
AG	36 (30.51)	97 (34.52)		0.81 (0.51–1.29)	0.377	0.82 (0.51–1.32)	0.415
GG	5 (4.24)	16 (5.69)		0.68 (0.24–1.93)	0.470	0.70 (0.25–1.98)	0.499
Additive			0.565	0.82 (0.56–1.19)	0.288	0.83 (0.57–1.20)	0.323
Dominant	41 (34.75)	113 (40.21)	0.306	0.79 (0.51–1.24)	0.306	0.80 (0.51–1.26)	0.343
Recessive	113 (95.76)	265 (94.31)	0.552	0.73 (0.26–2.05)	0.554	0.74 (0.26–2.09)	0.575

^a^*χ*^*2*^ test for genotype distributions between neuroblastoma patients and controls.

^b^Adjusted for age and gender.

### *LMO1* polymorphisms and neuroblastoma risk in combined subjects

To further elucidate the association between *LMO1* polymorphisms and neuroblastoma risk, we combined our present results the data from our earlier study [22]. In the combination analysis (Table 2), we found that those carrying the rs110419 G allele were at decreased risk of neuroblastoma (AG vs. AA: adjusted OR = 0.67, 95% CI = 0.51–0.88, *P* = 0.004; GG vs. AA: adjusted OR = 0.58, 95% CI = 0.40–0.84, *P* = 0.004; AG/GG vs. AA: adjusted OR = 0.65, 95% CI = 0.50–0.83, *P* = 0.001), as were those carrying the rs4758051 A allele (AA vs. GG: adjusted OR = 0.57, 95% CI = 0.39–0.84, *P* = 0.005; AA vs. GG/AG: adjusted OR = 0.59, 95% CI = 0.41–0.84, *P* = 0.004) or the rs10840002 G allele (GG vs. AA: adjusted OR = 0.66, 95% CI = 0.46–0.95, *P* = 0.026; GG vs. AG/AA: adjusted OR = 0.68, 95% CI = 0.49–0.94, *P* = 0.020). No significant association was observed between rs204938 A > G and neuroblastoma risk.

**Table 2:** *LMO1* polymorphisms and neuroblastoma susceptibility in combined subjects

Genotype	Cases (*N* = 374)	Controls (*N* = 812)	*P*^a^	Crude OR (95% CI)	*P*	Adjusted OR (95% CI)^b^	*P*^b^
rs110419 (HWE = 0.239)							
AA	150 (40.11)	245 (30.17)		1.00		1.00	
AG	171 (45.72)	417 (51.35)		**0.67 (0.51–0.88)**	**0.004**	**0.67 (0.51–0.88)**	**0.004**
GG	53 (14.17)	150 (18.47)		**0.58 (0.40–0.84)**	**0.004**	**0.58 (0.40–0.84)**	**0.004**
Additive			0.003	**0.74 (0.62–0.89)**	**0.001**	**0.74 (0.62–0.89)**	**0.001**
Dominant	224 (59.89)	567 (69.83)	0.001	**0.65 (0.50–0.83)**	**0.001**	**0.65 (0.50–0.83)**	**0.001**
Recessive	321 (85.83)	662 (81.53)	0.068	0.73 (0.52–1.02)	0.068	0.73 (0.52–1.03)	0.069
rs4758051 (HWE = 0.271)							
GG	145 (38.77)	282 (34.73)		1.00		1.00	
AG	185 (49.47)	380 (46.80)		0.95 (0.73–1.24)	0.688	0.95 (0.73–1.24)	0.698
AA	44 (11.76)	150 (18.47)		**0.57 (0.39–0.84)**	**0.005**	**0.57 (0.39–0.84)**	**0.005**
Additive			0.014	**0.80 (0.67–0.96)**	**0.014**	**0.80 (0.67–0.96)**	**0.014**
Dominant	229 (61.23)	530 (65.27)	0.178	0.84 (0.65–1.08)	0.178	0.84 (0.65–1.08)	0.182
Recessive	330 (88.24)	662 (81.53)	0.004	**0.59 (0.41–0.85)**	**0.004**	**0.59 (0.41–0.84)**	**0.004**
rs10840002 (HWE = 0.233)							
AA	132 (35.29)	260 (32.02)		1.00		1.00	
AG	186 (49.73)	384 (47.29)		0.95 (0.73–1.25)	0.736	0.96 (0.73–1.26)	0.744
GG	56 (14.97)	168 (20.69)		**0.66 (0.46–0.95)**	**0.025**	**0.66 (0.46–0.95)**	**0.026**
Additive			0.062	**0.83 (0.70–0.99)**	**0.042**	**0.83 (0.70–0.99)**	**0.043**
Dominant	242 (64.71)	552 (67.98)	0.265	0.86 (0.67–1.12)	0.266	0.87 (0.67–1.12)	0.270
Recessive	318 (85.03)	644 (79.31)	0.019	**0.68 (0.49–0.94)**	**0.020**	**0.68 (0.49–0.94)**	**0.020**
rs204938 (HWE = 0.485)							
AA	241 (64.44)	522 (64.29)		1.00		1.00	
AG	119 (31.82)	262 (32.27)		0.98 (0.76–1.28)	0.904	0.98 (0.76–1.28)	0.908
GG	14 (3.74)	28 (3.45)		1.08 (0.56–2.09)	0.813	1.07 (0.55–2.08)	0.834
Additive			0.961	1.01 (0.81–1.25)	0.967	1.00 (0.81–1.25)	0.976
Dominant	133 (35.56)	290 (35.71)	0.959	0.99 (0.77–1.28)	0.959	0.99 (0.77–1.28)	0.958
Recessive	360 (96.26)	784 (96.55)	0.798	1.09 (0.57–2.09)	0.798	1.08 (0.56–2.08)	0.820

^a^*χ*^*2*^ test for genotype distributions between neuroblastoma patients and controls.

^b^Adjusted for age and gender.

False-positive report probability (FPRP) analysis showed that all of the statistically significant associations remained noteworthy, when a prior probability of association of 0.25 was considered. At a prior probability level of 0.1, all except one association (rs10840002 A > G) remained noteworthy. At a prior probability level of 0.01, only the association between rs110419 A > G and neuroblastoma risk remained noteworthy (FPRP = 0.168). Detailed information from the FPRP analysis is listed in Table 3.

**Table 3:** False-positive report probability values for significant findings in combined subjects

Genotype	Crude OR (95% CI)	*P*^a^	Statistical Power^b^	Prior probability				
				0.25	0.1	0.01	0.001	0.0001
rs110419 A > G								
AG vs. AA	0.67 (0.51–0.88)	0.004	0.606	**0.017**	**0.051**	0.370	0.856	0.983
GG vs. AA	0.58 (0.40–0.84)	0.004	0.256	**0.044**	**0.120**	0.601	0.938	0.993
AG/GG vs. AA	0.65 (0.50–0.83)	0.001	0.392	**0.006**	**0.018**	**0.168**	0.671	0.953
rs4758051 G > A								
AA vs. GG	0.57 (0.39–0.84)	0.005	0.284	**0.050**	**0.137**	0.635	0.946	0.994
AA vs. GG/AG	0.59 (0.41–0.85)	0.004	0.259	**0.044**	**0.122**	0.605	0.939	0.994
rs10840002 A > G								
GG vs. AA	0.66 (0.46–0.95)	0.025	0.557	**0.119**	0.288	0.816	0.978	0.998
GG vs. AG/AA	0.68 (0.49–0.94)	0.020	0.521	**0.103**	0.257	0.792	0.975	0.997

^a^Chi-square test was used to calculate the genotype frequency distributions.

^b^Statistical power was calculated using the number of observations in the subgroup and the OR and *P* values in this table.

## DISCUSSION

In the present case-control study, we further verified the effect of *LMO1* polymorphisms on neuroblastoma risk in a Northern Chinese population. Consistent with our earlier findings [22], we observed that *LMO1* polymorphisms were associated with a decreased risk of neuroblastoma. To our knowledge, this is the first study to validate the association between *LMO1* polymorphisms and neuroblastoma risk using two resident groups in China. This combined analysis improves the statistical power for assessing the impact of *LMO1* polymorphisms on neuroblastoma risk.

A previous genome-wide association study revealed that *LMO1* polymorphisms were associated with predisposition to neuroblastoma [16]. In that study, Wang et al. detected four SNPs in *LMO1* gene (rs110419 A > G, rs4758051 G > A, rs10840002 A > G and rs204938 A > G) that were associated with neuroblastoma risk in subjects of European ancestry. Thereafter, case-control studies conducted with Italian [24], African-American [25] and Northern Chinese [26] populations verified *LMO1* polymorphisms to be factors affecting neuroblastoma risk. In 2016, we conducted the first epidemiological study on the effect of *LMO1* polymorphisms on neuroblastoma susceptibility in a Southern Chinese population [22]. We genotyped the aforementioned *LMO1* SNPs in 256 cases and 531 controls. We only found that the rs110419 A > G polymorphism was associated with a significantly lower neuroblastoma risk. However the strength of that conclusion was limited by the small sample. We have therefore now expanded the size of our sample.

Here, we assessed the relationship between *LMO1* polymorphisms and neuroblastoma risk in an additional 118 cases and 281 controls. Our analysis indicates that carrying the rs4758051 A or rs10840002 G allele is associated with decreased risk of neuroblastoma in a Northern Chinese population. In our earlier study of a Southern Chinese population, we detected a significant protective association between only the rs110419 A > G polymorphism and neuroblastoma risk [22]. Two possible explanations for this discrepancy are as follows. First, the small sizes of the samples used in these two studies means the statistical power of analyzing the association between a single polymorphism and cancer risk is small. Second, because these two studies were conducted in different regions in China, the inconsistency may be attributable to differences in the genetic variations, environmental exposures, and gene-environment interactions across the different regions.

To increase the representation for our conclusions, we combined the results from our present study with those from our earlier one. The combined analysis indicated that carrying the rs110419 G, rs4758051 A or rs10840002 G allele was associated with a decreased risk for neuroblastoma. The larger sample in the combined analysis highlights the important protective effect of *LMO1* polymorphisms on neuroblastoma risk. In addition, the FPRP analysis also enhances the robustness of our findings.

Although this is a relatively large sample for investigating the correlation between *LMO1* polymorphisms and neuroblastoma risk in the Chinese, several limitations still exist. First, we only genotyped four SNPs in this study, other potentially functional polymorphisms not discovered in genome-wide association studies were omitted. These include the rs2168101 G > T polymorphism, which was recently found to be associated with neuroblastoma [14]. Second, all of the subjects were recruited from two hospitals and most lived in Southern or Northern China, which inevitably caused selection bias. Third, we failed to assess several important environmental factors, including dietary intake, paternal exposures, and the subjects’ living environment. The absence of such information limits our ability to conduct a gene-environmental interaction analysis. Fourth, the sample size is still not large enough to ensure a robust conclusion. Fifth, the potential mechanisms of action of the four polymorphisms were not studied. Experimental analysis of the mechanisms of potentially functional *LMO1* polymorphisms is needed.

In summary, we have further confirmed the protective effect of *LMO1* polymorphisms on neuroblastoma susceptibility in a Chinese population. More case-control studies based on other ethnicities and multicenter investigations are encouraged to support these observations.

## MATERIALS AND METHODS

### Study subjects

The recruitment criteria for neuroblastoma patients and controls were described previously [22, 27, 28]. A total of 118 neuroblastoma patients and 281 healthy controls from Henan province (Northern China) were ultimately included in the study [29]. Briefly, all children with neuroblastoma histologically confirmed at The First Affiliated Hospital of Zhengzhou University between August 2011 and April 2017 were enrolled in the study. During the same period, 281 age- and gender-matched controls were also recruited at the same hospital. Before their participation, we obtained informed written consent for all subjects. The present study was approved by the Institutional Review Board of the hospital.

### SNP selection and genotyping

Four *LMO1* SNPs (rs110419 A > G, rs4758051 G > A, rs10840002 A > G and rs204938 A > G) identified as being associated with neuroblastoma in an earlier genome-wide association study were selected (Supplementary Table 2) [16]. Genotyping these four SNPs was performed using Taqman real-time PCR. The detailed procedure can be found in our earlier study [30]. To ensure the accuracy of the genotyping results, about 10% of the samples were also genotyped by sequencing [31, 32], and 100% genotype concordance was obtained.

### Statistical analysis

The goodness-of-fit χ^2^ test was applied to assess whether the selected SNPs were in Hardy-Weinberg equilibrium among the controls. Two-sided χ^2^ tests were employed to compare demographic variables and genotype frequencies between cases and controls. To evaluate the strength of the relationship between *LMO1* polymorphisms and neuroblastoma susceptibility, ORs and 95% CIs were calculated using unconditional logistic regression analyses. To determine whether the significant findings were “noteworthy”, we adopted the FPRP analysis [33, 34]. We calculated FPRP for a range of prior probabilities from 0.0001 to 0.25 and used 0.2 as a cut-point for FPRP. Values of *P* < 0.05 were considered statistically significant. SAS software (version 9.4; SAS Institute, Cary, NC) was used to perform all statistical analyses.

## SUPPLEMENTARY MATERIALS TABLES


