# Supplementary material for: LMO1 polymorphisms reduce neuroblastoma risk in Chinese children: a two-center case-control study
Source: Oncotarget. 2017 Aug 7;8(39):65620–6. doi: 10.18632/oncotarget.20018 (PMC5630358; doi:10.18632/oncotarget.20018)
Supplement: Supplementary file 1 [file oncotarget-08-65620-s001.pdf]

LMO1 polymorphisms reduce neuroblastoma risk in Chinese children: a two-center case-control study

SUPPLEMENTARY MATERIALS

Supplementary Table 1: Frequency distribution of selected characteristics in neuroblastoma patients and controls

Variables	Cases (*n* = 118)		Controls (*n* = 281)		*P*[a]
	No.	%	No.	%	
Age range, month	0–131.1		0.1–144.0		0.189
Mean ± SD	46.24 ± 29.98		44.97 ± 33.23		
≤ 18	23	19.49	72	25.62	
> 18	95	80.51	209	74.38	
Gender					0.196
Female	54	45.76	109	38.79	
Male	64	54.24	172	61.21	
Clinical stage					
I	15	12.82			
II	31	26.50			
III	19	16.24			
IV	49	41.88			
4 s	3	2.56			
Site of origin					
Adrenal gland	89	75.42			
Mediastinum	19	16.10			
Other region	10	8.47			

[a]Two-sided X^2 test for distributions between neuroblastoma patients and controls.

Supplementary Table 2: Information on the four *LMO1* polymorphisms predicted by SNPinfo software

rs	Chromosome	Position	Region	Allele	TFBS	Splicing	miRNA	Allele	Asian	CHB
rs110419	11	8209429	intron	A/G	—	—	—	A	0.634	0.53
rs4758051	11	8195215	intron	A/G	—	—	—	G	0.528	0.518
rs10840002	11	8199602	intron	A/G	—	—	—	A	0.500	0.47
rs204938	11	8234773	intron	T/C	—	—	—	C	0.211	0.179

TFBS, transcription factor binding sites; CHB, Han Chinese in Beijing, China.
